# Potential Diagnostic Role for a Combined Postmortem DNA and RNA Sequencing for Brugada Syndrome

**DOI:** 10.1161/CIRCGEN.122.004251

**Published:** 2023-10-05

**Authors:** Carlos Bueno-Beti, David C. Johnson, Chris Miles, Joseph Westaby, Mary N. Sheppard, Elijah R. Behr, Angeliki Asimaki

**Affiliations:** 1 Cardiovascular Clinical Academic Group, Molecular and Clinical Research Science Institute, St George’s University of London & St George’s University Hospital NHS Foundation Trust, London, United Kingdom.

**Keywords:** autopsy, Brugada syndrome, diagnosis, genetic testing, sodium

Postmortem genetic testing (molecular autopsy) is an important tool to identify genetic risk in family members following an unexplained sudden death (sudden arrhythmic death syndrome). However, exome sequencing is currently informative in only 13% to 30% of cases.^1^ RNA sequencing (RNAseq) has been shown to aid genetic diagnosis where DNA sequencing (DNAseq) is uninformative. Formalin-fixed paraffin-embedded (FFPE) heart tissue is retained routinely for histopathologic examination after sudden cardiac death. Unfortunately, FFPE processing can lead to fragmentation, DNA crosslinks, and deamination leading to false positives variant calling in the subsequent sequencing. Brugada Syndrome (BrS), a heritable arrhythmia syndrome, is the most common underlying cause of death in sudden arrhythmic death syndrome.^1^ One gene, sodium voltage-gated channel alpha subunit 5 (*SCN5A*), has definitive evidence for disease causation but only underlies ≈20% of clinical cases,^2^ hampering the potential role of molecular autopsy as a diagnostic tool. We aimed to demonstrate that the combination of the DNAseq and RNAseq in postmortem tissue can successfully identify putative causative variants and establish whether there may be a distinctive functional expression profile in the right ventricular outflow tract of BrS decedents.

Six BrS cases with an antemortem diagnosis retrieved from the medical record employing expert consensus and Shanghai scoring criteria,^3^ and 5 age- and sex-matched controls with anon-cardiac death were selected for this study. FFPE heart tissue from the RVOT, and where available, suitable samples of splenic tissue, were obtained from the Cardiac Risk in the Young Center for Cardiac Pathology at St George’s, University of London. DNA and RNA were extracted from the FFPE heart samples following manufacturer’s instructions, and their integrity was determined with Agilent Tape Station. DNAseq and RNAseq were undertaken on an Illumina HiSeq instrument. Sequence adapters were removed from 2×150 paired-end RNA sequencing with Trimmomatic v0.39. Alignment was undertaken with STAR-2.7.3a onGRCh38. Quality control metrics were assessed by FastQC, QualiMap, RNASeqMetrics, and PICARD. FeatureCounts generated counts for each gene. To call variants, SplitNCigarReads, BaseRecalibrator, ApplyBQSR, and HaplotypeCaller were applied to aligned DNAseq and RNAseq in accordance with germline short variant discovery GATK (v4) guidance. Overall,198 unique genes were investigated including sudden cardiac death (n=87), BrS (n=23), and trusight cardio (n=172). Differential expression between BrS cases and Control subjects was assessed using DeSeq2.27 (false discovery rate cutoff at 0.01). Gene set enrichment analysis was performed using all genes ranked by their differential mRNA expression Ethical approval was granted by the London Stanmore National Health Service Research Ethics Committee (reference: 10/H0724/38).

Variant calling on DNAseq data revealed 2 *SCN5A* variants, p.S1315X in case B4 and p.T17I in case B6 classified as pathogenic and likely pathogenic, respectively, by American College of Medical Genetics criteria. Additionally, we observed 19 variants of uncertain significance in 14 different genes (Genome Aggregation Database allele frequency ≤10^-4^, popmax filtering allele frequency <1.85×10^-4^ and reads ≥20; Figure).

**Figure.**
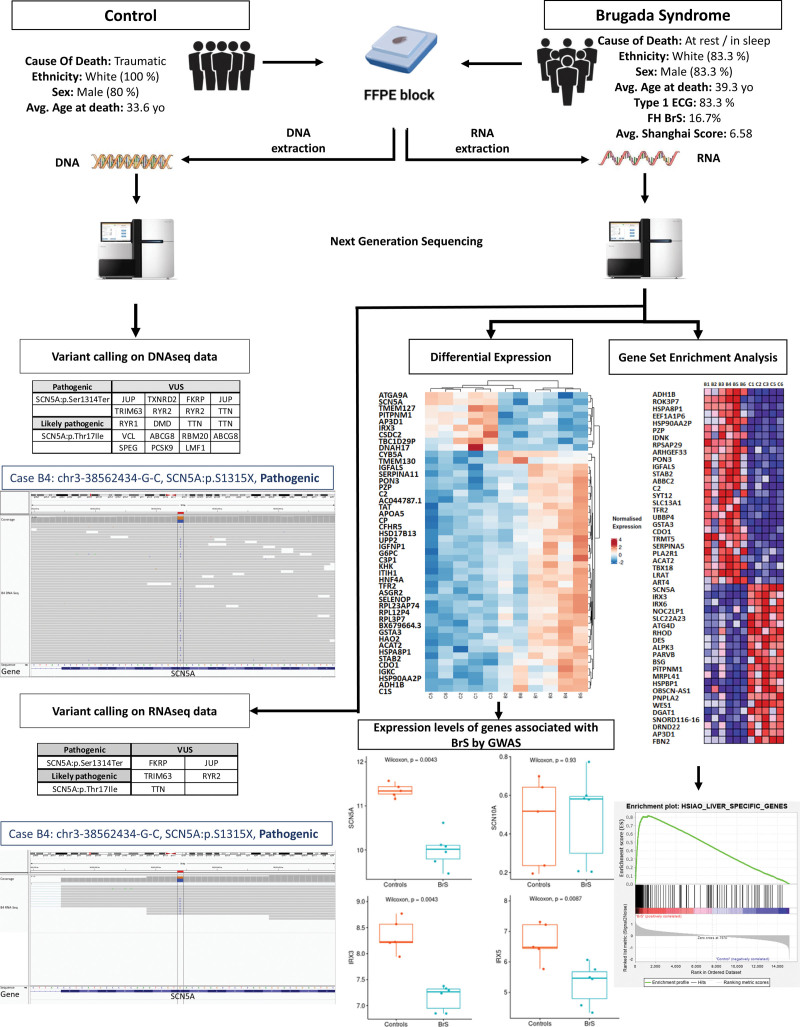
**Combined DNA-sequencing (DNAseq) and RNA-sequencing (RNAseq) analysis approach as a potential diagnostic tool in Brugada syndrome (BrS).** Formalin-fixed paraffin-embedded (FFPE) heart tissue from the right ventricular outflow tract from 6 patients with BrS and 5 age- and sex-matched controls with a noncardiac death, were selected for this study. DNA and RNA from all samples were extracted and sequenced on an Illumina HiSeq instrument. Alignment was undertaken with STAR-2.7.3a on GRCh38. Quality control metrics were assessed by FastQC, QualiMap, RNASeqMetrics, and PICARD. FeatureCounts generated counts for each gene. To call variants, SplitNCigarReads, BaseRecalibrator, ApplyBQSR, and HaplotypeCaller were applied to aligned DNAseq and RNAseq in accordance with germline short variant discovery GATK (v4) guidance. A total of 198 genes were investigated. Differential expression between BrS cases and Control subjects was assessed using DeSeq2.27 (false discovery rate cutoff at 0.01). Gene set enrichment analysis was performed using all genes ranked by their differential mRNA expression. FH indicates family history; *IRX3*, Iroquois Homeobox 3; *IRX5*, Iroquois Homeobox 5; *SCN5A*, sodium voltage-gated channel alpha subunit 5; and VUS, variants of uncertain significance.

Variant calling on RNAseq data (Genome Aggregation Database allele frequency ≤10^-5^, CADD >20, and RNA reads ≥20) confirmed the presence of *SCN5A*: p.S1315X and *SCN5A*: p.T17I in the transcriptome. Only 5 out of the 19 VUSs in genes *FKRP*, *JUP*, *TRIM63*, *RYR2*, and *TTN* identified in DNAseq data were detected in the RNAseq data set. The subjects were heterozygous for all the variants identified in this study. Three genes at loci previously demonstrated as genome-wide significant associated with BrS, *SCN5A*, Iroquois Homeobox 3 (*IRX3*) , and Iroquois Homeobox 5 (*IRX5*), showed significant differential expression between BrS and controls, regardless of the presence of a *SCN5A* variant (Figure).

Gene set expression analysis revealed 50 novel genetic associations with BrS. Interestingly,13 of these 50 new associations are present in 2 liver-specific gene sets (mean normalized enrichment score=4.06 and 3.30 with false discovery rate=0; Figure).

The data that support the findings of this study are available from the corresponding author on reasonable request.

By merging variant calling data from DNAseq with RNA-seq from the same tissue source, an improved diagnostic accuracy of variant calling can be achieved in molecular autopsy. Gene expression analysis of RNAseq data from FFPE heart samples demonstrated reduced SCN5A expression levels in all BrS patients, regardless of *SCN5A* genotype. Furthermore, we associated 50 novel genes, including liver-specific gene sets, with BrS that could be used as an expression profile of the disease with the potential for improving the diagnostic accuracy and yield in sudden arrhythmic death syndrome decedents. Interestingly, the most strongly associated liver gene, alcohol dehydrogenase 1B (*ADH1B*), has been previously associated with arrhythmic events after alcohol drinking in a BrS cohort.^4^

The transcriptional and post-transcriptional regulation of SCN5A in myocardial tissue may determine the penetrance and expressivity of associated diseases such as BrS. IRX3 and IRX5, 2 well-known regulators of the expression of different ion channels in the adult heart showed reduced expression in BrS decedents. *SCN5A*, *IRX3*, and *IRX5* have proximal SNP variants associated with BrS with genome-wide significance, suggesting that the regulation of these genes is important in BrS risk.^5^

This study, therefore, supports the potential utility of combining RNAseq with DNAseq of FFPE tissue of sudden cardiac death decedents in a novel approach to molecular autopsy that requires further prospective investigation. It also unveils genomic pathways adding to the risk of BrS.

## ARTICLE INFORMATION

### Sources of Funding

Drs Behr and Johnson are funded by St George’s Hospital Charity, RES 19-20 002 “Genomics in Sudden Cardiac Death and Inherited Cardiac Conditions.” Dr Behr is supported by the Robert Lancaster Memorial Fund. Dr Miles was the recipient of a British Heart Foundation clinical research training fellowship (FS/18/28/33549). Drs Sheppard and Westaby, are supported by the charity, Cardiac Risk in the Young (CRY). Dr Asimaki is supported by the Rosetrees Foundation Trust corn seed fund (M689), the British Heart Foundation project grant (PG/18/27/33616), and the Wellcome Trust project grant (208460/Z/17/Z). Dr Bueno-Beti is supported by the British Heart Foundation project grant (PG/18/27/33616). This research was funded in whole, or in part, by the Wellcome Trust (Grant number 208460/Z/17/Z). For the purpose of open access, the authors have applied a Creative Commons Attribution (CC BY) license to any Author Accepted Manuscript (AAM) version arising from this submission.

### Disclosures

Dr Behr has undertaken consulting for Boston Scientific in the last 3 years. The other authors report no conflicts.
